# A new polymorph of 1,3-bis­(penta­fluoro­phen­yl)urea

**DOI:** 10.1107/S1600536813007836

**Published:** 2013-03-28

**Authors:** Tsunehisa Okuno

**Affiliations:** a Department of Material Science and Chemistry, Wakayama University, Sakaedani, Wakayama, 640-8510, Japan

## Abstract

The title compound, C_13_H_2_F_10_N_2_O, has been previously described in the space group *Pbca* with *Z* = 8 [Jai-nhuknan *et al.* (1997 ▶). *Acta Cryst.* C**53**, 455–457]. The current *P*2_1_2_1_2_1_ polymorph was obtained from a tetra­hydro­furan solution. The penta­fluoro­phenyl rings make dihedral angles of 50.35 (6) and 54.94 (6)° with the urea fragment, in close accord with those reported for the first polymorph. In the crystal, both of the N—H groups donate H atoms to the same carbonyl O atom, forming a one-dimensional mol­ecular array along the *a* axis. There are close contacts between perfluoro­phenyl C atoms within the array [3.228 (3) Å] and halogen bonds are also observed between the arrays [F⋯F = 2.709 (2) and 2.7323 (18) Å].

## Related literature

For the structure of the first reported ploymorph, see: Jai-nhuknan *et al.* (1997 ▶). For the related structure of 1,3-diphenyl­urea, see: Dannecker *et al.* (1979 ▶). For background to organofluorine chemistry, see: Chambers (2004 ▶).
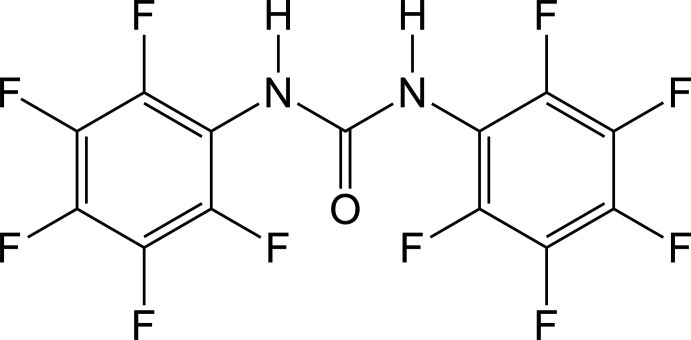


## Experimental

### 

#### Crystal data

C_13_H_2_F_10_N_2_O
*M* *_r_* = 392.16
Orthorhombic, 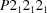

*a* = 4.5798 (7) Å
*b* = 9.5411 (16) Å
*c* = 29.136 (5) Å
*V* = 1273.1 (4) Å^3^
*Z* = 4
Mo *K*α radiation
μ = 0.23 mm^−1^
*T* = 93 K
0.15 × 0.15 × 0.03 mm

#### Data collection

Rigaku Saturn724+ diffractometer
Absorption correction: numerical (*NUMABS*; Rigaku, 1999 ▶) *T*_min_ = 0.978, *T*_max_ = 0.993
10307 measured reflections
1993 independent reflections
1941 reflections with *F* ^2^ > 2σ(*F* ^2^)
*R*_int_ = 0.023

#### Refinement

*R*[*F* ^2^ > 2σ(*F* ^2^)] = 0.031
*wR*(*F* ^2^) = 0.080
*S* = 1.09
1991 reflections
241 parameters
2 restraints
Only H-atom coordinates refined
Δρ_max_ = 0.34 e Å^−3^
Δρ_min_ = −0.20 e Å^−3^

### 

Data collection: *CrystalClear* (Rigaku, 2008 ▶); cell refinement: *CrystalClear*; data reduction: *CrystalClear*; program(s) used to solve structure: *SIR92* (Altomare, *et al.*, 1994 ▶); program(s) used to refine structure: *SHELXL97* (Sheldrick, 2008 ▶); molecular graphics: *ORTEP-3 for Windows* (Farrugia, 2012 ▶); software used to prepare material for publication: *CrystalStructure* (Rigaku, 2010 ▶).

## Supplementary Material

Crystal structure: contains datablock(s) global, I. DOI: 10.1107/S1600536813007836/ff2101sup1.cif

Structure factors: contains datablock(s) I. DOI: 10.1107/S1600536813007836/ff2101Isup2.hkl

Supplementary material file. DOI: 10.1107/S1600536813007836/ff2101Isup3.cml

Additional supplementary materials:  crystallographic information; 3D view; checkCIF report

## Figures and Tables

**Table 1:** Hydrogen-bond geometry (Å, °)

*D*—H⋯*A*	*D*—H	H⋯*A*	*D*⋯*A*	*D*—H⋯*A*
N1—H1⋯O1^i^	0.864 (18)	2.057 (18)	2.850 (3)	152 (3)
N2—H2⋯O1^i^	0.879 (18)	2.008 (18)	2.825 (3)	154 (3)

Symmetry code: (i) 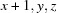.

**Fig. 1.**
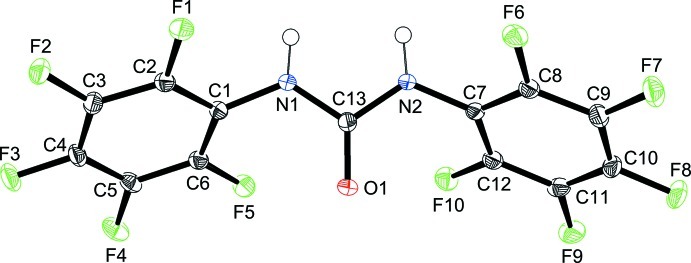
The asymmetric unit of the title compound with atom-numbering scheme. Displacement ellipsoids are drawn at the 50% probability level and H atoms are shown as small spheres.

**Fig. 2.**
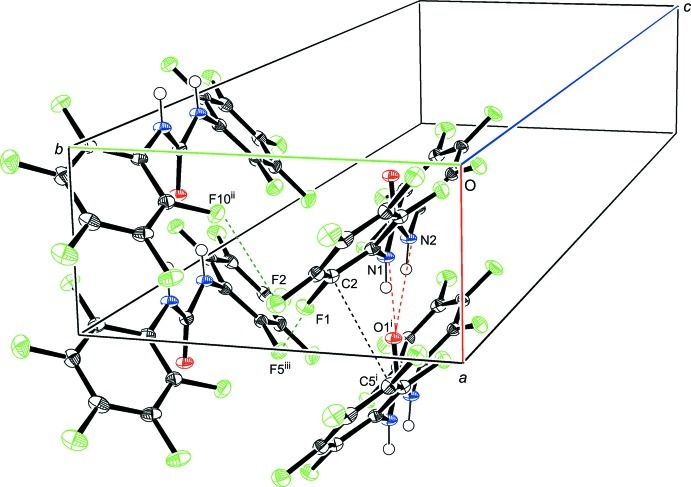
A view of the intermolecular interactions in the title compound. [Symmetry codes: (i) x + 1, y, z (ii) -x + 1, y + 1/2, -z + 1/2 (iii)-x + 2, y + 1/2, -z + 1/2.]

## supplementary crystallographic information

### Comment

Perfluoro aromatic compounds have attracted interest from the viewpoint of the electronic and structural comparison with the original compounds (Chambers, 2004). The title compound, (I), is a perfluoro compound of 1,3-diphenylurea (Dannecker *et al.*, 1979). Previously, (I) was isolated in an orthorhombic *Pbca* polymorph with *Z* = 8 [Jai-nhuknan *et al.*, 1997]. A new orthorhombic *P*2_1_2_1_2_1_ polymorph was obtained by recrystallization from a tetrahydrofuran solution.

The dihedral angle between the C1—C6/F1—F5 (r.m.s. deviation = 0.0223 Å) and the C7—C12/F6—F10 (r.m.s. deviation = 0.0181 Å) pentafluorophenyl rings is 30.93 (3)°, which is smaller than that of the reported polymorph. The pentafluorophenyl rings make dihedral angles of 50.35 (6)° and 54.94 (6)°, respectively, with the C13/N1/N2/O1 urea fragment (r.m.s. deviation = 0.0011 Å), and this situation is almost accordance with the reported polymorph.

Both of the N—H groups in one molecule donate protons to the same carbonyl O atom, forming a one-dimensional molecular array along the *a* axis, where the N···O distances were 2.850 (3) Å for N1···O1^i^ and 2.825 (3) Å for N2···O1^i^ [Symmetry code: (i) *x* + 1, *y*, *z*] (Figure 2). There are close contacts between perfluorophenyl carbons within the array, where the C2···C5^i^ distance is 3.228 (3) Å. Halogen-bonds are also recognized between the arrays. The distances of F2···F10^ii^ and F1···F5^iii^ are 2.709 (2) Å and 2.7323 (18) Å, respectively [Symmetry codes: (ii) -*x* + 1, *y* + 1/2, -*z* + 1/2 (iii)-*x* + 2, *y* + 1/2, -*z* + 1/2.] (Figure 2).

### Experimental

The title compound was commercially purchased and recrystallized from a tetrahydrofuran solution.

### Refinement

Friedel pairs were merged because the molecule itself was achiral and because there were not any anomalous scattering effects. The N-bound H atom was obtained from a difference Fourier map and was refined isotropically with the restriction of N—H range between 0.85 Å and 0.89 Å. *U*_iso_(H) values of the H atoms were set at 1.2*U*_eq_(parent atom).

### Crystal data

**Table:**

C_13_H_2_F_10_N_2_O	*F*(000) = 768.00
*M**_r_* = 392.16	*D*_x_ = 2.046 Mg m^−^^3^
Orthorhombic, *P*2_1_2_1_2_1_	Mo *K*α radiation, λ = 0.71075 Å
Hall symbol: P 2ac 2ab	Cell parameters from 4650 reflections
*a* = 4.5798 (7) Å	θ = 2.3–31.2°
*b* = 9.5411 (16) Å	µ = 0.23 mm^−^^1^
*c* = 29.136 (5) Å	*T* = 93 K
*V* = 1273.1 (4) Å^3^	Platelet, colorless
*Z* = 4	0.15 × 0.15 × 0.03 mm

### Data collection

**Table:**

Rigaku Saturn724+ diffractometer	1941 reflections with *F*^2^ > 2σ(*F*^2^)
Detector resolution: 28.445 pixels mm^-1^	*R*_int_ = 0.023
ω scans	θ_max_ = 29.0°
Absorption correction: numerical (*NUMABS*; Rigaku, 1999)	*h* = −6→5
*T*_min_ = 0.978, *T*_max_ = 0.993	*k* = −12→13
10307 measured reflections	*l* = −39→37
1993 independent reflections	

### Refinement

**Table:**

Refinement on *F*^2^	Secondary atom site location: difference Fourier map
*R*[*F*^2^ > 2σ(*F*^2^)] = 0.031	Hydrogen site location: inferred from neighbouring sites
*wR*(*F*^2^) = 0.080	Only H-atom coordinates refined
*S* = 1.09	*w* = 1/[σ^2^(*F*_o_^2^) + (0.0393*P*)^2^ + 0.6807*P*] where *P* = (*F*_o_^2^ + 2*F*_c_^2^)/3
1991 reflections	(Δ/σ)_max_ < 0.001
241 parameters	Δρ_max_ = 0.34 e Å^−^^3^
2 restraints	Δρ_min_ = −0.20 e Å^−^^3^
Primary atom site location: structure-invariant direct methods	

### Special details

**Table:**

Geometry. ENTER SPECIAL DETAILS OF THE MOLECULAR GEOMETRY
Refinement. Refinement was performed using all reflections except for two with very negative *F*^2^. The weighted *R*-factor (*wR*) and goodness of fit (*S*) are based on *F*^2^. *R*-factor (gt) are based on *F*. The threshold expression of *F*^2^ > 2.0 σ(*F*^2^) is used only for calculating *R*-factor (gt).

### Fractional atomic coordinates and isotropic or equivalent isotropic displacement parameters (Å^2^)

**Table:**

	*x*	*y*	*z*	*U*_iso_*/*U*_eq_	
F1	1.1418 (3)	0.61873 (13)	0.20307 (4)	0.0201 (3)	
F2	0.9729 (3)	0.60115 (14)	0.11485 (4)	0.0230 (3)	
F3	0.5296 (4)	0.42251 (14)	0.09057 (4)	0.0261 (3)	
F4	0.2849 (3)	0.25518 (13)	0.15527 (4)	0.0235 (3)	
F5	0.4741 (3)	0.26032 (12)	0.24218 (4)	0.0199 (3)	
F6	1.1205 (3)	0.65289 (13)	0.41004 (4)	0.0209 (3)	
F7	0.9048 (4)	0.66890 (14)	0.49612 (4)	0.0269 (3)	
F8	0.4722 (4)	0.49024 (15)	0.52340 (4)	0.0286 (3)	
F9	0.2803 (3)	0.28817 (15)	0.46530 (4)	0.0261 (3)	
F10	0.5071 (3)	0.26629 (12)	0.38011 (4)	0.0213 (3)	
O1	0.4972 (3)	0.46701 (15)	0.30935 (5)	0.0163 (3)	
N1	0.9286 (4)	0.4477 (2)	0.27009 (5)	0.0154 (3)	
N2	0.9324 (4)	0.45284 (19)	0.34806 (5)	0.0149 (3)	
C1	0.8112 (5)	0.4427 (2)	0.22555 (6)	0.0143 (4)	
C2	0.9298 (5)	0.5288 (2)	0.19172 (6)	0.0156 (4)	
C3	0.8433 (5)	0.5207 (2)	0.14616 (6)	0.0172 (4)	
C4	0.6225 (5)	0.4295 (3)	0.13398 (6)	0.0180 (4)	
C5	0.4991 (5)	0.3442 (2)	0.16699 (6)	0.0170 (4)	
C6	0.5947 (5)	0.3492 (2)	0.21201 (6)	0.0150 (4)	
C7	0.8117 (4)	0.4624 (2)	0.39231 (6)	0.0141 (4)	
C8	0.9103 (5)	0.5630 (2)	0.42307 (6)	0.0155 (4)	
C9	0.8013 (5)	0.5723 (2)	0.46721 (6)	0.0181 (4)	
C10	0.5852 (5)	0.4809 (3)	0.48127 (6)	0.0196 (4)	
C11	0.4853 (5)	0.3785 (2)	0.45158 (6)	0.0184 (4)	
C12	0.6005 (5)	0.3694 (2)	0.40767 (6)	0.0158 (4)	
C13	0.7652 (5)	0.4563 (2)	0.30915 (6)	0.0139 (4)	
H1	1.117 (4)	0.451 (3)	0.2722 (9)	0.0184*	
H2	1.123 (4)	0.459 (3)	0.3452 (9)	0.0179*	

### Atomic displacement parameters (Å^2^)

**Table:**

	*U*^11^	*U*^22^	*U*^33^	*U*^12^	*U*^13^	*U*^23^
F1	0.0170 (6)	0.0229 (6)	0.0204 (6)	−0.0048 (5)	−0.0003 (5)	−0.0012 (5)
F2	0.0234 (6)	0.0297 (6)	0.0158 (5)	0.0024 (6)	0.0033 (5)	0.0049 (5)
F3	0.0296 (7)	0.0353 (7)	0.0135 (5)	0.0054 (7)	−0.0075 (6)	−0.0045 (5)
F4	0.0220 (6)	0.0210 (6)	0.0276 (6)	−0.0030 (6)	−0.0094 (6)	−0.0061 (5)
F5	0.0200 (6)	0.0195 (6)	0.0202 (6)	−0.0027 (6)	−0.0016 (5)	0.0030 (5)
F6	0.0185 (6)	0.0226 (6)	0.0215 (6)	−0.0072 (6)	−0.0018 (5)	0.0029 (5)
F7	0.0326 (8)	0.0299 (7)	0.0181 (5)	−0.0002 (7)	−0.0049 (6)	−0.0073 (5)
F8	0.0303 (7)	0.0426 (8)	0.0129 (5)	0.0047 (7)	0.0060 (6)	0.0026 (5)
F9	0.0201 (6)	0.0324 (7)	0.0259 (6)	−0.0066 (6)	0.0043 (6)	0.0104 (6)
F10	0.0221 (6)	0.0197 (6)	0.0221 (6)	−0.0056 (6)	−0.0011 (5)	−0.0017 (5)
O1	0.0100 (6)	0.0234 (7)	0.0154 (6)	−0.0001 (6)	−0.0011 (6)	0.0006 (6)
N1	0.0097 (7)	0.0242 (8)	0.0122 (7)	0.0003 (7)	−0.0006 (6)	−0.0007 (6)
N2	0.0094 (7)	0.0237 (8)	0.0117 (7)	0.0000 (7)	−0.0004 (6)	0.0004 (6)
C1	0.0126 (8)	0.0174 (8)	0.0129 (8)	0.0016 (8)	−0.0009 (7)	−0.0011 (7)
C2	0.0134 (8)	0.0178 (8)	0.0155 (8)	0.0009 (7)	−0.0004 (7)	−0.0017 (7)
C3	0.0180 (9)	0.0202 (9)	0.0135 (8)	0.0046 (8)	0.0014 (7)	0.0013 (7)
C4	0.0193 (9)	0.0230 (9)	0.0116 (8)	0.0063 (9)	−0.0036 (8)	−0.0040 (7)
C5	0.0159 (9)	0.0165 (8)	0.0186 (8)	0.0001 (8)	−0.0051 (8)	−0.0043 (7)
C6	0.0136 (8)	0.0163 (8)	0.0152 (8)	0.0015 (8)	−0.0007 (7)	−0.0010 (7)
C7	0.0111 (8)	0.0184 (9)	0.0127 (8)	0.0013 (8)	−0.0003 (7)	0.0026 (7)
C8	0.0134 (8)	0.0180 (8)	0.0151 (8)	−0.0005 (8)	−0.0008 (7)	0.0026 (7)
C9	0.0197 (9)	0.0200 (9)	0.0147 (8)	0.0027 (8)	−0.0029 (8)	−0.0017 (7)
C10	0.0190 (9)	0.0277 (10)	0.0122 (8)	0.0060 (9)	0.0015 (7)	0.0032 (7)
C11	0.0141 (8)	0.0224 (9)	0.0188 (8)	0.0003 (8)	0.0015 (8)	0.0081 (7)
C12	0.0139 (8)	0.0172 (8)	0.0162 (8)	0.0003 (8)	−0.0012 (7)	0.0010 (7)
C13	0.0141 (8)	0.0150 (8)	0.0126 (8)	−0.0006 (7)	−0.0003 (7)	0.0000 (7)

### Geometric parameters (Å, º)

**Table:**

F1—C2	1.337 (3)	C1—C2	1.393 (3)
F2—C3	1.332 (3)	C1—C6	1.391 (3)
F3—C4	1.336 (3)	C2—C3	1.387 (3)
F4—C5	1.342 (3)	C3—C4	1.380 (3)
F5—C6	1.340 (3)	C4—C5	1.381 (3)
F6—C8	1.344 (3)	C5—C6	1.384 (3)
F7—C9	1.336 (3)	C7—C8	1.389 (3)
F8—C10	1.335 (3)	C7—C12	1.387 (3)
F9—C11	1.336 (3)	C8—C9	1.382 (3)
F10—C12	1.340 (3)	C9—C10	1.381 (3)
O1—C13	1.232 (3)	C10—C11	1.383 (3)
N1—C1	1.405 (3)	C11—C12	1.387 (3)
N1—C13	1.364 (3)	N1—H1	0.864 (18)
N2—C7	1.406 (3)	N2—H2	0.879 (18)
N2—C13	1.368 (3)		
			
F1···F2	2.6895 (18)	F9···C11^xiv^	3.197 (3)
F1···N1	2.725 (2)	F10···F1^vi^	3.2313 (18)
F2···F3	2.744 (2)	F10···F2^viii^	2.709 (2)
F3···F4	2.7126 (18)	F10···F2^vi^	2.859 (2)
F4···F5	2.6767 (18)	F10···F3^viii^	3.3936 (19)
F5···O1	2.7803 (19)	F10···N2^v^	3.312 (3)
F5···N1	2.862 (3)	F10···C3^viii^	2.942 (3)
F5···C13	3.014 (3)	F10···C4^viii^	3.293 (3)
F6···F7	2.6999 (18)	O1···F4^i^	3.101 (2)
F6···N2	2.765 (2)	O1···F5^i^	3.1785 (19)
F7···F8	2.732 (2)	O1···N1^v^	2.850 (3)
F8···F9	2.712 (2)	O1···N2^v^	2.825 (3)
F9···F10	2.6989 (18)	O1···C13^v^	3.354 (3)
F10···O1	2.8143 (19)	N1···F1^vi^	3.251 (3)
F10···N2	2.799 (3)	N1···F5^iii^	3.178 (3)
F10···C13	2.993 (3)	N1···F5^i^	3.525 (3)
O1···C1	2.843 (3)	N1···O1^iii^	2.850 (3)
O1···C6	3.083 (3)	N2···F1^vi^	3.535 (3)
O1···C7	2.814 (3)	N2···F2^vi^	3.552 (3)
O1···C12	3.049 (3)	N2···F4^i^	3.053 (3)
C1···C4	2.807 (3)	N2···F10^iii^	3.312 (3)
C2···C5	2.741 (3)	N2···O1^iii^	2.825 (3)
C2···C13	3.571 (3)	C1···F1^v^	3.557 (3)
C3···C6	2.767 (3)	C1···F4^iii^	3.478 (3)
C6···C13	3.109 (3)	C1···F5^iii^	3.532 (3)
C7···C10	2.797 (3)	C1···F5^i^	3.432 (3)
C8···C11	2.753 (3)	C2···F4^iii^	3.254 (3)
C8···C13	3.535 (3)	C2···F5^i^	3.466 (3)
C9···C12	2.757 (3)	C2···C5^iii^	3.228 (3)
C12···C13	3.082 (3)	C2···C6^iii^	3.544 (3)
F1···F5^i^	3.5106 (18)	C3···F4^iii^	3.252 (3)
F1···F5^ii^	2.7323 (18)	C3···F10^i^	2.942 (3)
F1···F10^ii^	3.2313 (18)	C3···C5^iii^	3.496 (3)
F1···N1^ii^	3.251 (3)	C4···F1^v^	3.487 (3)
F1···N2^ii^	3.535 (3)	C4···F2^v^	3.441 (3)
F1···C1^iii^	3.557 (3)	C4···F4^iii^	3.515 (3)
F1···C4^iii^	3.487 (3)	C4···F6^vi^	3.162 (3)
F1···C5^iii^	3.263 (3)	C4···F10^i^	3.293 (3)
F1···C6^iii^	3.315 (3)	C5···F1^v^	3.263 (3)
F1···C6^ii^	3.523 (3)	C5···F6^vi^	3.377 (3)
F1···C13^ii^	3.269 (3)	C5···C2^v^	3.228 (3)
F2···F3^iii^	3.147 (2)	C5···C3^v^	3.496 (3)
F2···F8^iv^	2.8148 (18)	C6···F1^v^	3.315 (3)
F2···F9^i^	3.1594 (19)	C6···F1^vi^	3.523 (3)
F2···F10^i^	2.709 (2)	C6···C2^v^	3.544 (3)
F2···F10^ii^	2.859 (2)	C7···F2^vi^	3.591 (3)
F2···N2^ii^	3.552 (3)	C7···F4^i^	3.150 (3)
F2···C4^iii^	3.441 (3)	C7···F9^iii^	3.448 (3)
F2···C7^ii^	3.591 (3)	C8···F4^i^	3.061 (3)
F2···C12^ii^	3.286 (3)	C8···F9^iii^	3.356 (3)
F3···F2^v^	3.147 (2)	C8···C11^iii^	3.275 (3)
F3···F6^vi^	3.0309 (19)	C9···F7^xi^	3.246 (3)
F3···F7^iv^	2.9024 (18)	C9···F8^iii^	3.568 (3)
F3···F8^vii^	3.131 (2)	C9···F9^iii^	3.487 (3)
F3···F8^iv^	3.119 (2)	C10···F6^v^	3.395 (3)
F3···F10^i^	3.3936 (19)	C10···F7^xi^	3.504 (3)
F4···F6^viii^	2.8319 (18)	C10···F9^xiii^	3.133 (3)
F4···F6^vi^	3.4627 (19)	C11···F6^v^	3.333 (3)
F4···O1^viii^	3.101 (2)	C11···F9^xiii^	3.197 (3)
F4···N2^viii^	3.053 (3)	C11···C8^v^	3.275 (3)
F4···C1^v^	3.478 (3)	C12···F2^vi^	3.286 (3)
F4···C2^v^	3.254 (3)	C12···F6^v^	3.486 (3)
F4···C3^v^	3.252 (3)	C13···F1^vi^	3.269 (3)
F4···C4^v^	3.515 (3)	C13···F4^i^	3.043 (3)
F4···C7^viii^	3.150 (3)	C13···F5^i^	3.442 (3)
F4···C8^viii^	3.061 (3)	C13···O1^iii^	3.354 (3)
F4···C13^viii^	3.043 (3)	F1···H1	2.58 (3)
F5···F1^viii^	3.5106 (18)	F5···H1	3.57 (2)
F5···F1^vi^	2.7323 (18)	F6···H2	2.64 (3)
F5···O1^viii^	3.1785 (19)	F10···H2	3.52 (3)
F5···N1^v^	3.178 (3)	O1···H1	3.040 (19)
F5···N1^viii^	3.525 (3)	O1···H2	3.052 (19)
F5···C1^v^	3.532 (3)	N1···H2	2.37 (3)
F5···C1^viii^	3.432 (3)	N2···H1	2.37 (3)
F5···C2^viii^	3.466 (3)	C2···H1	2.61 (3)
F5···C13^viii^	3.442 (3)	C6···H1	3.12 (3)
F6···F3^ii^	3.0309 (19)	C8···H2	2.66 (3)
F6···F4^i^	2.8319 (18)	C12···H2	3.13 (3)
F6···F4^ii^	3.4627 (19)	H1···H2	2.13 (4)
F6···F7^ix^	3.4730 (18)	F1···H1^ii^	3.46 (3)
F6···C4^ii^	3.162 (3)	F4···H2^viii^	3.39 (3)
F6···C5^ii^	3.377 (3)	F5···H1^v^	2.60 (3)
F6···C10^iii^	3.395 (3)	F5···H1^vi^	3.52 (3)
F6···C11^iii^	3.333 (3)	F10···H2^v^	2.74 (3)
F6···C12^iii^	3.486 (3)	O1···H1^v^	2.06 (2)
F7···F3^x^	2.9024 (18)	O1···H2^v^	2.01 (2)
F7···F6^xi^	3.4730 (18)	C1···H1^v^	3.460 (19)
F7···F7^xi^	2.773 (2)	C6···H1^v^	2.97 (3)
F7···F7^ix^	2.773 (2)	C7···H2^v^	3.44 (2)
F7···F8^iii^	3.208 (3)	C11···H2^v^	3.60 (3)
F7···F8^ix^	3.316 (2)	C12···H2^v^	2.97 (3)
F7···C9^ix^	3.246 (3)	C13···H1^v^	3.160 (19)
F7···C10^ix^	3.504 (3)	C13···H2^v^	3.123 (19)
F8···F2^x^	2.8148 (18)	H1···F1^vi^	3.46 (3)
F8···F3^xii^	3.131 (2)	H1···F5^iii^	2.60 (3)
F8···F3^x^	3.119 (2)	H1···F5^ii^	3.52 (3)
F8···F7^v^	3.208 (3)	H1···O1^iii^	2.06 (2)
F8···F7^xi^	3.316 (2)	H1···C1^iii^	3.460 (19)
F8···F9^xiii^	3.026 (2)	H1···C6^iii^	2.97 (3)
F8···C9^v^	3.568 (3)	H1···C13^iii^	3.160 (19)
F9···F2^viii^	3.1594 (19)	H2···F4^i^	3.39 (3)
F9···F8^xiv^	3.026 (2)	H2···F10^iii^	2.74 (3)
F9···F9^xiv^	3.1405 (19)	H2···O1^iii^	2.01 (2)
F9···F9^xiii^	3.1405 (19)	H2···C7^iii^	3.44 (2)
F9···C7^v^	3.448 (3)	H2···C11^iii^	3.60 (3)
F9···C8^v^	3.356 (3)	H2···C12^iii^	2.97 (3)
F9···C9^v^	3.487 (3)	H2···C13^iii^	3.123 (19)
F9···C10^xiv^	3.133 (3)		
			
C1—N1—C13	124.23 (17)	F6—C8—C7	119.45 (16)
C7—N2—C13	122.57 (16)	F6—C8—C9	118.75 (17)
N1—C1—C2	118.94 (18)	C7—C8—C9	121.79 (19)
N1—C1—C6	123.82 (17)	F7—C9—C8	120.16 (19)
C2—C1—C6	117.09 (17)	F7—C9—C10	120.19 (17)
F1—C2—C1	119.10 (16)	C8—C9—C10	119.65 (18)
F1—C2—C3	118.66 (17)	F8—C10—C9	120.58 (18)
C1—C2—C3	122.19 (18)	F8—C10—C11	119.58 (19)
F2—C3—C2	119.74 (18)	C9—C10—C11	119.84 (17)
F2—C3—C4	120.90 (16)	F9—C11—C10	120.09 (17)
C2—C3—C4	119.36 (18)	F9—C11—C12	120.21 (17)
F3—C4—C3	120.55 (18)	C10—C11—C12	119.70 (19)
F3—C4—C5	119.96 (19)	F10—C12—C7	119.93 (16)
C3—C4—C5	119.49 (17)	F10—C12—C11	118.52 (18)
F4—C5—C4	119.68 (17)	C7—C12—C11	121.54 (18)
F4—C5—C6	119.63 (17)	O1—C13—N1	123.73 (17)
C4—C5—C6	120.70 (19)	O1—C13—N2	123.75 (17)
F5—C6—C1	120.90 (16)	N1—C13—N2	112.52 (17)
F5—C6—C5	118.00 (17)	C1—N1—H1	116.7 (17)
C1—C6—C5	121.09 (18)	C13—N1—H1	119.0 (17)
N2—C7—C8	120.58 (17)	C7—N2—H2	118.2 (17)
N2—C7—C12	121.92 (17)	C13—N2—H2	118.4 (17)
C8—C7—C12	117.45 (17)		
			
C1—N1—C13—O1	−3.5 (3)	F4—C5—C6—F5	2.1 (3)
C1—N1—C13—N2	177.01 (17)	F4—C5—C6—C1	−178.37 (15)
C13—N1—C1—C2	133.29 (19)	C4—C5—C6—F5	−177.65 (17)
C13—N1—C1—C6	−51.4 (3)	C4—C5—C6—C1	1.8 (3)
C7—N2—C13—O1	−0.5 (3)	N2—C7—C8—F6	0.8 (3)
C7—N2—C13—N1	179.00 (16)	N2—C7—C8—C9	−178.16 (16)
C13—N2—C7—C8	−125.94 (19)	N2—C7—C12—F10	0.4 (3)
C13—N2—C7—C12	56.8 (3)	N2—C7—C12—C11	179.16 (16)
N1—C1—C2—F1	−3.3 (3)	C8—C7—C12—F10	−176.99 (16)
N1—C1—C2—C3	174.14 (16)	C8—C7—C12—C11	1.8 (3)
N1—C1—C6—F5	3.0 (3)	C12—C7—C8—F6	178.23 (16)
N1—C1—C6—C5	−176.48 (16)	C12—C7—C8—C9	−0.8 (3)
C2—C1—C6—F5	178.43 (16)	F6—C8—C9—F7	−0.1 (3)
C2—C1—C6—C5	−1.1 (3)	F6—C8—C9—C10	179.98 (15)
C6—C1—C2—F1	−178.95 (16)	C7—C8—C9—F7	178.88 (17)
C6—C1—C2—C3	−1.5 (3)	C7—C8—C9—C10	−1.0 (3)
F1—C2—C3—F2	0.4 (3)	F7—C9—C10—F8	1.7 (3)
F1—C2—C3—C4	−179.26 (15)	F7—C9—C10—C11	−178.09 (17)
C1—C2—C3—F2	−177.03 (17)	C8—C9—C10—F8	−178.41 (18)
C1—C2—C3—C4	3.3 (3)	C8—C9—C10—C11	1.8 (3)
F2—C3—C4—F3	−1.5 (3)	F8—C10—C11—F9	−1.1 (3)
F2—C3—C4—C5	177.88 (16)	F8—C10—C11—C12	179.42 (16)
C2—C3—C4—F3	178.15 (17)	C9—C10—C11—F9	178.72 (18)
C2—C3—C4—C5	−2.5 (3)	C9—C10—C11—C12	−0.8 (3)
F3—C4—C5—F4	−0.4 (3)	F9—C11—C12—F10	−1.8 (3)
F3—C4—C5—C6	179.35 (16)	F9—C11—C12—C7	179.43 (16)
C3—C4—C5—F4	−179.83 (18)	C10—C11—C12—F10	177.76 (17)
C3—C4—C5—C6	−0.0 (3)	C10—C11—C12—C7	−1.0 (3)

Symmetry codes: (i) −*x*+1, *y*+1/2, −*z*+1/2; (ii) −*x*+2, *y*+1/2, −*z*+1/2; (iii) *x*+1, *y*, *z*; (iv) −*x*+3/2, −*y*+1, *z*−1/2; (v) *x*−1, *y*, *z*; (vi) −*x*+2, *y*−1/2, −*z*+1/2; (vii) −*x*+1/2, −*y*+1, *z*−1/2; (viii) −*x*+1, *y*−1/2, −*z*+1/2; (ix) *x*+1/2, −*y*+3/2, −*z*+1; (x) −*x*+3/2, −*y*+1, *z*+1/2; (xi) *x*−1/2, −*y*+3/2, −*z*+1; (xii) −*x*+1/2, −*y*+1, *z*+1/2; (xiii) *x*+1/2, −*y*+1/2, −*z*+1; (xiv) *x*−1/2, −*y*+1/2, −*z*+1.

### Hydrogen-bond geometry (Å, º)

**Table:**

*D*—H···*A*	*D*—H	H···*A*	*D*···*A*	*D*—H···*A*
N1—H1···O1^iii^	0.864 (18)	2.057 (18)	2.850 (3)	152 (3)
N2—H2···O1^iii^	0.879 (18)	2.008 (18)	2.825 (3)	154 (3)

Symmetry code: (iii) *x*+1, *y*, *z*.
